# Neo-Adjuvant Use of Sorafenib for Hepatocellular Carcinoma Awaiting Liver Transplantation

**DOI:** 10.3389/ti.2022.10569

**Published:** 2022-11-09

**Authors:** Kate Minoux, Guillaume Lassailly, Massih Ningarhari, Henri Lubret, Medhi El Amrani, Valérie Canva, Stéphanie Truant, Philippe Mathurin, Alexandre Louvet, Gilles Lebuffe, Odile Goria, Eric Nguyen-Khac, Emmanuel Boleslawski, Sebastien Dharancy

**Affiliations:** ^1^ CHU Lille, Department of Hepatogastroenterology, Lille, France; ^2^ INSERM U995, University of Lille, Lille, France; ^3^ CHU Lille, Department of Digestive Surgery and Transplantation, University of Lille, Lille, France; ^4^ CHU Lille, Department of Anesthesiology, Resuscitation, and Critical Care Anesthesiology, University of Lille, Lille, France; ^5^ CHU Rouen, Department of Hepatogastroenterology, Hôpital Charles Nicolle, Rouen, France; ^6^ CHU Amiens-Picardie, Hôpital Sud, Department of Hepatogastroenterology, Amiens, France; ^7^ CHU Amiens, Centre Universitaire de Recherche en Santé (CURS), Université de Picardie-Jules-Verne (UPJV), Groupe de Recherche sur l’alcool et les Pharmacodépendances (GRAP), Inserm U1247, Amiens, France

**Keywords:** liver transplantation, cancer, hepatocellular carcinoma (HCC), cancer-therapeutics, sorafenib, neoadjuvant therapy, tyrosine kinase inhibitor

## Abstract

Data on efficacy and safety of sorafenib in a neoadjuvant setting for HCC awaiting liver transplantation (LT) are heterogeneous and scarce. We aimed to investigate the trajectory of patients treated with sorafenib while awaiting LT. All patients listed for HCC and treated with sorafenib were included in a monocentric observational study. A clinical and biological evaluation was performed every month. Radiological tumor response evaluation was realized every 3 months on the waiting list and every 6 months after LT. Among 327 patients listed for HCC, 62 (19%) were treated with Sorafenib. Sorafenib was initiated for HCC progression after loco-regional therapy (LRT) in 50% of cases and for impossibility of LRT in 50% of cases. The mean duration of treatment was 6 months. Thirty six patients (58%) dropped-out for tumor progression and 26 (42%) patients were transplanted. The 5-year overall and recurrent-free survival after LT was 77% and 48% respectively. Patients treated for impossibility of LRT had acceptable 5-year intention-to-treat overall and post-LT survivals. Conversely, patients treated for HCC progression presented high dropout rate and low intention-to-treat survival. Our results suggest that it is very questionable in terms of utility that patients treated for HCC progression should even be kept listed once the tumor progression has been observed.

## Graphical Abstract

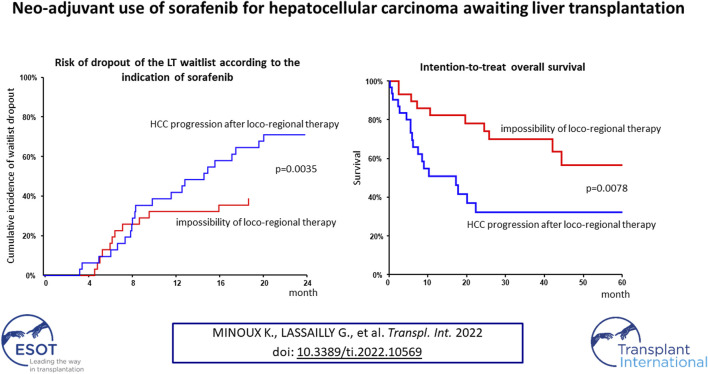

## Introduction

Liver Transplantation (LT) is the only therapy that, unlike other curative treatments (ablative therapies, surgical resection), simultaneously cures hepatocellular carcinoma (HCC) and the underlying liver disease. However, very few patients are eligible for LT because of their condition (age, comorbidities), behavior (observance, abstinence in alcohol consumption) and tumor biology and spread. The eligibility of LT in our country is based on the alpha-fetoprotein (AFP) score which includes the number of nodules, their size, and the AFP level (1). According to the French agency for organ allocation (Agence de la Biomédecine), HCC is currently the leading indication for LT in France, accounting for 30% of registrations on the waiting list. The dropout nor shortage imposes a waiting time before LT which may lead to tumor progression beyond accepted criteria.

Strategies to minimize or avoid waitlist dropout related to tumor progression include loco-regional therapy (LRT). Indeed, transarterial modalities (transarterial chemoembolization—TACE, transarterial radioembolization—TARE) and percutaneous thermal ablative strategies (radio frequency ablation—RFA, microwave ablation) have been widely adopted by transplant programs to bridge HCC candidates before LT. A consensus statement for LT for HCC has recommended LRT if the anticipated waiting time for an organ to become available exceeds 6 months (2). By limiting the risk of progression on the waiting list, LRT also reduces the risk of recurrence after LT, especially when a partial or complete response according to mRECIST is achieved before LT (3–5). Other prognostic factors such as low AFP level, low number of tumor nodules and small total tumor diameter at baseline, extended post-interventional tumor necrosis, well differentiated tumor grade and lack of microvascular invasion have been shown to reduce post-LT HCC recurrence (6). Tumor recurrence is the main cause of mortality after LT for HCC with a 5-year survival of 22% in case of recurrence (7). It is therefore crucial to optimize management of patients awaiting LT to improve their long-term prognosis.

Sorafenib is a multikinase inhibitor with activity against both the tumor cell directly (inhibition of cell proliferation, notably through the Raf signaling pathway) and the endothelial cells of blood vessels (inhibition of angiogenesis through the VEGF and PDGF signaling pathway) (8). It was the first systemic therapy to prolong survival in patients with advanced HCC, suggesting that its use in the neoadjuvant setting may be beneficial (9). However, there remains a concern that sorafenib’s anti-angiogenic effect may interfere with tissue repair-healing and thus lead to increased post-LT complications. Data on efficacy and safety of sorafenib in this setting are heterogeneous and scarce so far (10–17).

We sought to analyze in a large cohort of patients treated with sorafenib as neoadjuvant therapy for HCC: 1) Trajectories of patients awaiting LT treated with sorafenib (Intention-to-treat survival, dropout rate, tolerance, radiological response to treatment), 2) peri-operative morbidity and 3) overall (OS) and recurrence-free survival after LT.

## Patients and Methods

### Study Characteristics and Population

This is a single-center, non-randomized and observational study. We included all candidate to LT for HCC patients listed between May 2010 and April 2019 and treated with sorafenib for at least 1 day on the waiting list. Patients were identified thanks to the nationwide CRISTAL registry. Diagnosis of HCC was established by pathological analysis of directed biopsies or according to the non-invasive criteria of the European Association for the Study of the Liver (EASL) guidelines (18, 19). Each indication of LT was submitted to validation of a multidisciplinary liver committee, which included at least a liver surgeon, a hepatologist, an oncologist and a radiologist specialized in HCC and LT.

**FIGURE 1:**
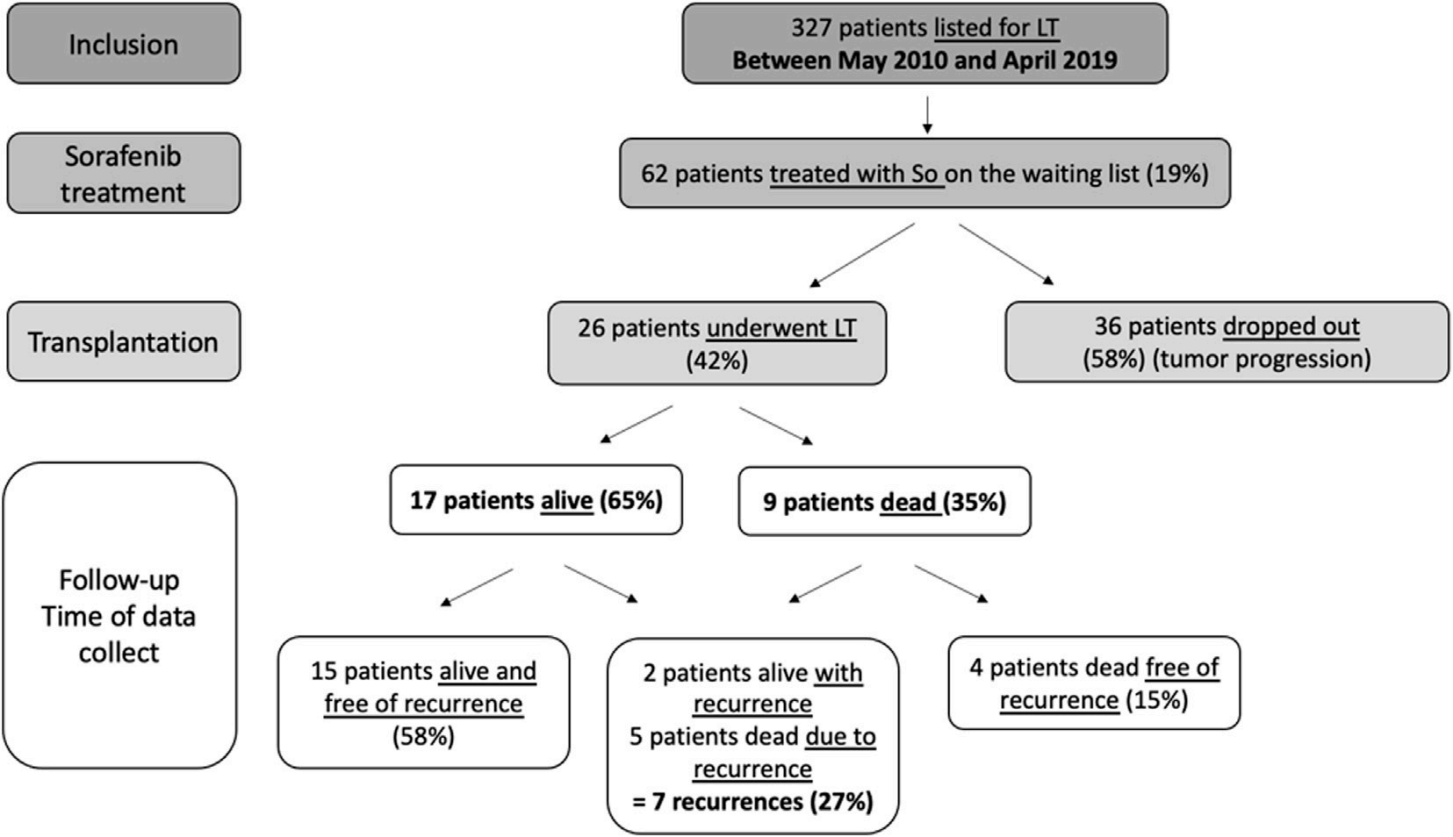
Flow chart.

All patients had measurable disease parameters that had been classified according to mRECIST (modified Response Evaluation Criteria in Solid Tumours) with no evidence of radiologically definable major vascular invasion or extrahepatic metastases. Study flow chart is presented in Figure 1. In accordance with French law, all patients were informed that their medical information could be used for non-interventional research purposes (according to the Jardé law).

### Indication and Management of Sorafenib

Sorafenib was used on-label after validation by multidisciplinary liver conference. It was initiated in two different cases: in case of tumor progression after failure of all types of LRT, or in case of impossibility of another LRT (multifocal tumor or technical impossibility). The technical impossibility and/or contraindication of another LRT has always been retained during a multidisciplinary committee considering all the available therapeutic alternatives. Main contraindication of TACE were arterio-portal shunt and portal vein thrombosis. In some cases low hypervascularity of HCC and/or multifocal small tumors (diameter < 2 cm) were the main drawbacks. Main contraindications of percutaneous thermal ablative strategies were the presence of ascites on imagery and some location such as hepatic dome.

Sorafenib was mainly introduced to prevent dropout but could also be introduced in few cases (*n* = 4) to try tumor down-staging by reducing tumor burden for patients initially outside eligibility criteria (AFP score > 2). In terms of trajectory, such patients who had been putt on the waiting list and treated with sorafenib had to present partial/complete response and/or a decrease in AFP level to allow being transplanted.

Patients started treatment either at 400 mg twice a day (full dose) or at 200 mg twice a day with escalation at full dose in case of good liver function and absence of side effects.

### Follow-Up Awaiting Liver Transplantation

Liver transplant waiting list time was defined as the number of days from the time of activation on the liver transplant waiting list until the day of transplantation. Physical examination, adverse events and laboratory monitoring including biochemical and hematological parameters were carried out every month. Laboratory-based MELD and AFP score were calculated at each visit. Dose modifications, temporary treatment pauses, and symptomatic treatments were prescribed depending on side effects which were graded using the National Cancer Institute’s Common Terminology Criteria for adverse events. In case of a grade 2 adverse event, treatment was reduced to half dose and the patient was reassessed on day 15. In case of a grade 3 side effect, treatment was discontinued. Treatment was continued until the day of transplantation or until tumor progression.

Contrast-enhanced CT-scan or MRI was performed at baseline and repeated every 3 months. Radiological tumor response during treatment with sorafenib was assessed according to mRECIST (1). Complete response (CR) was defined as the absence of arterially enhanced areas in all target lesions; partial response (PR) and progressive disease (PD) as a greater than 30% decrease and a greater than 20% increase, respectively, in the sum of the longest diameters of arterial enhanced areas in all target lesions; and stable disease (SD) as neither PR nor PD. Radiological assessment of tumor characteristics (number of nodules, maximum nodule diameter and sum of all diameters) was collected retrospectively on last imaging preceding sorafenib introduction and on final pretransplant or prior to dropout imaging.

### Explant Histopathology Examination

All liver explants were examined by an experienced hepato-pathologist. Tumor characteristics, gross appearance (nodular or infiltrative), extent of tumor necrosis, vascular invasion, cell differentiation and presence of satellite nodules were analyzed.

### Peri-Operative Morbidity and Follow-Up

Peri-operative complications including incidences of surgical revision, sepsis, hemorrhage, vascular thrombosis, overall bile duct complication and bile duct stenosis, asymptomatic CMV infection, pathologically confirmed acute cellular rejection and re-transplantation were reported. Blood loss until the first month after LT and length of patient’s hospital stay were collected. Occurrences of HCC tumor recurrence after LT and OS were also identified.

Post-transplant monitoring was adapted to date of LT and included 6-monthly contrast-enhanced CT-scan or MRI imaging coupled with AFP measurements during the first 5 years of follow-up, then annually during 5 additional years. The database was fixed on March 2021 for the last news.

### Statistical Analysis

Demographic (age, gender), clinical (underlying liver disease, type of LRT preceding listing, waiting list time), carcinologic (AFP score), laboratory (MELD-score, AFP level and AFP score at listing), explant tumor characteristics and radiologic variables (tumor characteristics, Milan criteria) were registered. HCC recurrence free survival events were censored at the date of death or HCC recurrence. Continuous variables were summarized as means and standard deviation (SD) or medians and interquartile range (IQR). Comparisons of categorical and continuous variables were performed using the Chi-square test and the Mann–Whitney U-test, respectively. OS and recurrence-free survival rates were determined according to the Kaplan-Meier method. Patient survival in different groups was compared using the log-rank test. Survivals were expressed as percentage and 95% confidence interval (CI). A univariate linear regression comparison has been performed to identify predictors of HCC recurrence. A *p* value of 0.05 or less was considered statistically significant. Cumulative incidences of waitlist dropout with LT as competing risk event and HCC recurrence after LT with death without recurrence as competing event have been performed. All statistical analyses were performed using NCSS version 9.

## Results

### Patient Characteristics at Listing

During the period of May 2010 to April 2019, 327 HCC candidates were listed for LT. Of these patients, 62 (19%) were treated with sorafenib awaiting LT, among them 26 (42%) underwent LT and 36 (58%) dropped-out from the waiting list for tumor progression (Figure 1). Patient main characteristics are presented in Table 1. The majority of patients were middle-aged men and had compensated alcohol-related cirrhosis. There were no significant differences in demographic characteristics or therapeutic management prior to listing among the 2 groups, transplanted group (LT) and dropout group.

**TABLE 1:** Patient characteristics at listing.

	Total cohort^a^ *n* = 62	LT *n* = 26	Dropout *n* = 36	*p*
Age (years)				
Mean ± SD	59 ± 7.9	57 ± 9.7	60.5 ± 5.9	0.2
Median (IQR range)	61.2 (57.3–63.3)	61 (54.8–62.3)	61.5 (57.7–64.4)	
Gender, M/F, n (%)	51 (82.3%)/11 (17.7%)	20 (76.9%)/6 (23.1%)	31 (86.1%)/5(13.9%)	0.3
Etiology of cirrhosis, n (%)				
Alcohol	50 (80.7%)	20 (76.9%)	30 (83.3%)	0.2
Viral	5 (8.1%)	1 (3.9%)	4 (11.1%)	
Metabolic	3 (4.8%)	2 (7.7%)	1 (%)	
Hemochromatosis	1 (1.6%)	1 (3.8%)	0	
PBC	1 (1.6%)	1 (3.8%)	0	
Non cirrhotic liver	2 (3.2%)	1 (3.8%)	1 (2.8%)	
MELD				
Mean ± SD	10 ± 3.9	9.9 ± 3.2	10.1 ± 4.4	0.7
Median (IQR range)	9 (7–12.25)	9 (7–13)	8.5 (6–12)	
Treatment before listing, n (%)				
None	14 (22.6%)	8 (30.8%)	6 (16.7%)	0.3
TACE alone	22 (35.5%)	8 (30.8%)	14 (38.9%)	
Surgery alone	8 (12.9%)	4 (15.4%)	4 (11.1%)	
RFA alone	6 (9.7%)	1 (3.9%)	5 (13.9%)	
Combinations				
2 procedures				0.6
3 procedures	8 (12.9%)	3 (11.5%)	5 (13.9%)	
4 procedures	3 (4.8%)	1 (3.9%)	2 (5.6%)	
	1 (1.7%)	1 (3.9%)	0	

^a^ No missing data.

### HCC Characteristics at Listing

HCC characteristics are presented in Table 2. Approximately one third of patients had one nodule, one third had two nodules and one third had at least three nodules. Patients who dropped-out of the waiting list tended to have a larger maximum tumor diameter than transplanted patients (29.5 vs. 22.9 mm, *p* = 0.08). Mean AFP-level was 47.4 ± 123 UI/L.

**TABLE 2:** HCC characteristics at listing.

	Total cohort^a^ *n* = 62	LT *n* = 26	Dropout *n* = 36	*p*
Tumor number				
Mean ± SD	2.2 ± 1.3	2.5 ± 1.5	2 ± 1	
Median (IQR range)	2 (1–3)	2 (1–3)	2 (1–2)	0.1
Maximum tumor diameter				
Mean ± SD (mm)	26.7 ± 16.5	22.9 ± 8.2	29.5 ± 20.2	0.08
Total tumor diameter				
Mean ± SD (mm)	46.4 ± 27.3	45.1 ± 22.1	47.4 ± 30.8	0.8
Number of nodules, n (%)				
1 nodule	21 (33.9%)	8 (30.8%)	13 (36.1%)	0.07
2 nodules	23 (37.1%)	6 (23.1%)	17 (47.2%)	
3 nodules	9 (14.5%)	7 (26.9%)	2 (5.6%)	
>3 nodules	9 (14.5%)	5 (19.2%)	4 (11.1%)	
Largest nodule, n (%)				
<30 mm	44 (71%)	20 (76.9%)	24 (66.7%)	0.3
≥30 mm	18 (29%)	6 (23.1%)	12 (33.3%)	
Unique tumor, n (%)				
≤30 mm	18 (29%)	6 (23.1%)	12 (33.3%)	0.4
>30 mm	3 (4.8%)	2 (7.7%)	1 (2.8%)	
AFP-level (UI/L):				
Mean ± SD	47.4 ± 123.7	50.7 ± 126.1	45.1 ± 123.7	0.1
Median (IQR range)	8 (4–25.5)	6 (4–14)	11 (5–30)	
Milan criteria fulfilled, n (%)				
Yes/No	43 (69.4%)/19(30.7%)	18 (69.2%)/8 (30.8%)	25 (69.4%)/11 (30.6%)	0.9
AFP score, n (%)				
0	38 (61.3%)	14 (53.9%)	24 (66.7%)	0.4
1	8 (12.9%)	3 (11.5%)	5 (13.9%)	
2	12 (19.4%)	8 (30.8%)	4 (11.1%)	
3	3 (4.8%)	1 (3.9%)	2 (5.6%)	
4	1 (1.6%)	0	1 (2.8%)	

^a^ No missing data.

### Patient Management on Waiting List

Treatment indication is presented in Table 3. Half of the total cohort started sorafenib for tumor progression and the other half started sorafenib because of impossibility of LRT. There was a significant difference between the two groups in terms of treatment indication. Most transplanted patients who dropped-out initiated treatment because of tumor progression. Mean and median waiting time were respectively 13 ± 4.5 and 12.5 months (IQR: 11–14.7) from listing to LT, and respectively 10.4 ± 5.4 and 8.3 months (IQR: 6.2–15) from listing to dropout or death.

**TABLE 3:** Tolerance and treatment management of sorafenib.

	N	Total cohort	LT	Dropout	*p*
Treatment indication, n (%)					
Tumor progression	62	31 (50%)	8 (30.8%)	23 (63.Z%)	**0.01**
Impossibility of LRT		31 (50%)	18 (69.2%)	13 (36.1%)	
Treatment withdrawal, n (%)	61	42 (71%)	8 (30.8%)	34 (97.1%)	**<0.0001**
Reason for withdrawal, n (%)	42				
Intolerance		5 (11.9%)	0	5 (14.7%)	**0.009**
Tumor progression		22 (52.4%)	1 (12.5%)	21 (61.8%)	
Hepatic decompensation		13 (31%)	6 (75%)	7 (20.6%)	
Fatigue		2 (4.8%)	1 (12.5%)	1 (%)	
Sorafenib treatment duration (months)	62				
Mean ± SD		6 ± 7	8 ± 10	4.6 ± 3	0.4
Median (IQR range)		4.5 (2.25–7)	4.9 (1.1–10.9)	4.15 (2.3–6.2)	
Median start dose (IQR range)	61	400 (400–800)	800 (400–800)	400 (400–800)	0.4
Dose reduction, n (%)	61	25 (41%)	11 (44%)	14 (38.9%)	0.7
Aggravation at 1 month after introduction n (%)	62	13 (21%)	5 (19.2%)	8 (22.2%)	0.8
Adverse events, n (%)	62				
HFS/skin injury		26 (41.9%)	13 (50%)	13 (36.1%)	0.3
Fatigue		13 (21%)	5 (19.2%)	8 (22.2%)	0.8
		2 (3.2%)	1 (3.9%)	1 (2.8%)	0.8
Hematological toxicity		10 (16.1%)	5 (19.2%)	5 (13.9%)	0.6
Liver decompensation		23 (37.1%)	13 (50%)	10 (27.8%)	0.07
Gastrointestinal disorders		4 (6.5%)	1 (3.9%)	3 (8.3%)	0.5
Digestive bleeding		2 (3.2%)	2 (7.7%)	0	0.09
Hypertension		1 (1.6%)	0	1 (2.8%)	0.4
Neuropathy					
Sorafenib at time of LT, n (%)	26	—	18 (69.2%)	—	NA

Sorafenib was discontinued in 71% of all patients, mainly for hepatic decompensation in the LT group and mainly for tumor progression in the dropout group. Sixty-nine % of the transplanted patients had continued sorafenib until LT. In the total cohort, sorafenib was initiated at a median dose of 400 mg (IQR: 400–800) and continued for a mean duration of 6 months, with no significant differences between the LT and the dropout group. Gastrointestinal disorders (mainly diarrhea) tended to be more frequent in the LT group than in the dropout group (*p* = 0.07).

### Radiologic Assessment Prior to Liver Transplantation or Dropout

Maximum mean and median tumor diameter prior to LT or dropout was significantly higher in the dropout group than in the LT group (*p* = 0.002). Last mRECIST radiological response prior to LT or dropout is detailed in Table 4. Of the total cohort, 48.4% achieved disease control and 11.3% achieved objective response. Disease control was achieved in 73% in the LT group and 30.6% in dropout group (*p* = 0.001).

**TABLE 4:** Tumor characteristics and last radiological tumor response prior to LT or dropout.

	N	Total cohort	LT	Dropout	*p*
Sum of largest diameters (LD) (mm)	58				
Mean ± SD		65 ± 43	52 ± 28	75 ± 50	0.1
Median (IQR range)		56 (33.5–92.5)	50 (30.5–70)	60 (35–127)	
Maximum tumor diameter (mm):	58				
Mean ± DS		32.7 ± 25	22.1 ± 11	41.3 ± 29	**0.002**
Median (IQR range)		25 (18–37)	20 (17–27)	35 (20–53)	
Last mRECIST radiological response, n (%)	62				
CR		1 (1.6%)	1 (3.9%)	0	**0.001**
PR		6 (9.7%)	6 (23.1%)	0	
SD		23 (37.1%)	12 (46.2%)	11 (30.6%)	
PD		32 (51.6%)	7 (26.9%)	25 (69.4%)	

### Intention-to-Treat Survival and Incidence Rate of Dropout

**FIGURE 2:**
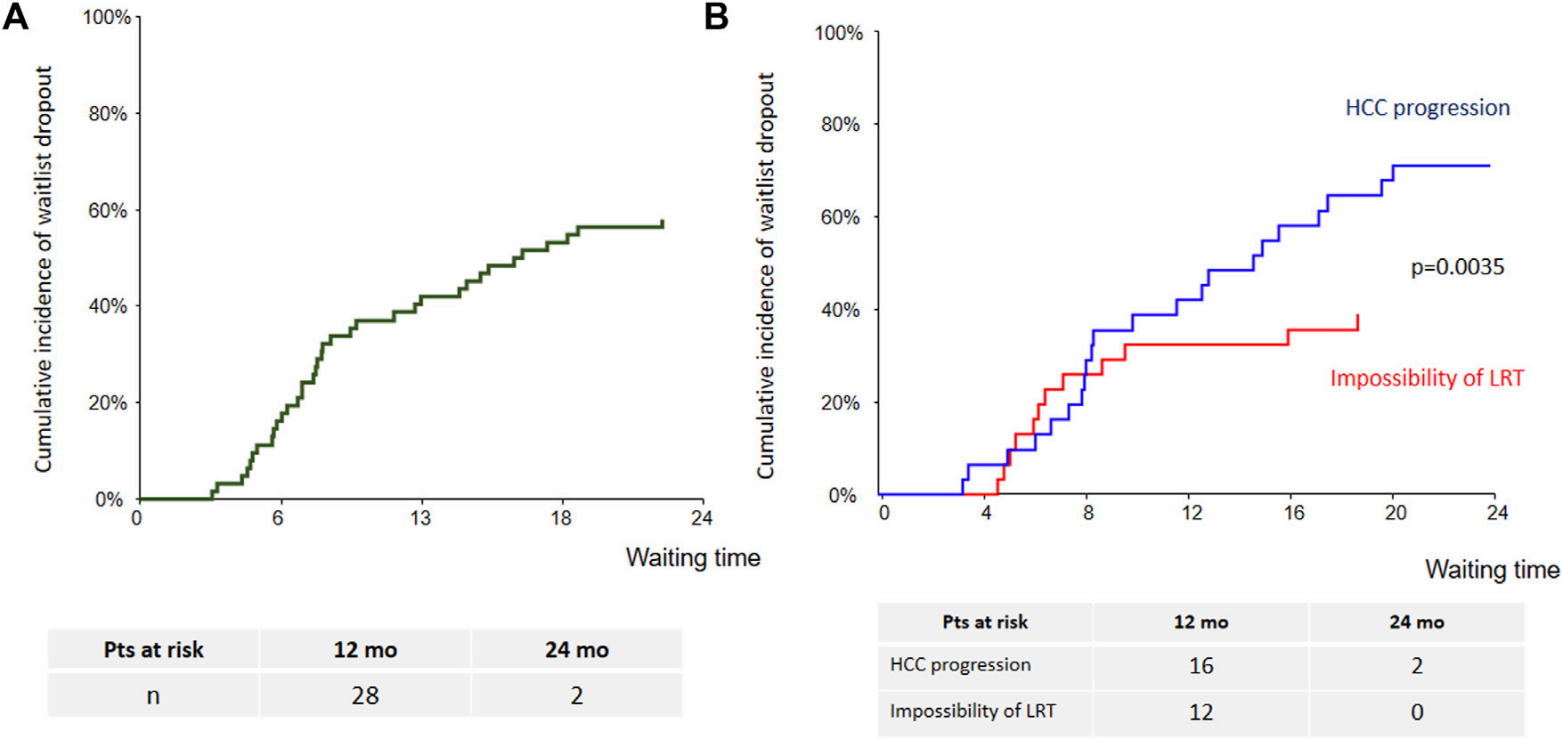
Cumulative incidence of waitlist dropout [**(A)**: whole cohort; **(B)**: according to sorafenib’s indication].

Cumulative incidence of waitlist dropout for the whole cohort is presented in Figure 2A. One- and 2-years dropout rates were 39% (95% CI: 28%–53%) and 56% (95% CI: 45%–70%). Patients treated with sorafenib for HCC progression had higher risk of dropout as compared with those treated for impossibility of LRT (*p* = 0.0035). At 1 year cumulative incidence rates of dropout were 32% (95% CI: 19%–53%) for impossibility of LRT and 42% for tumor progression (95% CI: 28%–63%) (Figure 2B). Among the four patients who were listed beyond eligibility criteria and treated with sorafenib in order to achieve tumor down-staging, only one have been transplanted.

**FIGURE 3:**
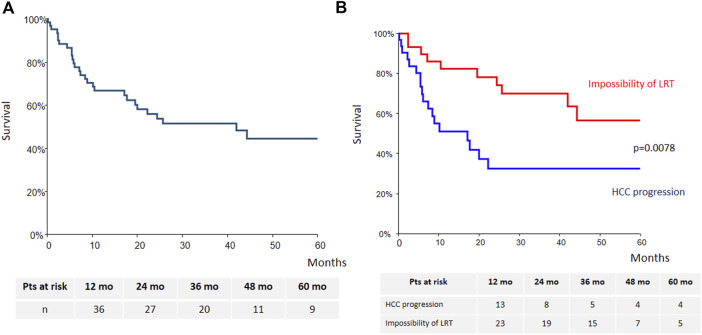
Intention-to-treat overall survival [**(A)**: whole cohort; **(B)**: according to sorafenib’s indication].

Intention-to-treat overall survival (OS) of the whole cohort is presented Figure 3A. Briefly, OS at years 1, 3 and 5 was 66% (95% CI: 54–79), 51.5% (95% CI: 38–65) and 44% (95% CI: 29%–59%), respectively. Patients treated with sorafenib for HCC progression had lower survival as compared with those treated for impossibility of LRT (*p* = 0.0078) (Figure 3B).

### Predictors of Dropout

We included discriminant factors associated with dropout in a logistic regression multivariable analysis. These factors were number of HCC at listing, maximal tumor diameter at listing, sorafenib’s indication and maximal tumor diameter at last radiological evaluation. Among them, sorafenib’s indication for tumor progression (Odds ratio 0.2, coefficient regression −1.5, *p* = 0.03) and maximal tumor diameter at last radiological evaluation (Odds ratio 1.08, coefficient regression 0.08, *p* = 0.006) were independent predictors of dropout.

### Explant Histopathology Analysis

Pathological examination exposed in Table 5 showed that most explants had ≥ 4 nodules (76%) which contained minimal necrosis (56.3%), no satellite nodules (75%) and no microvascular (80%) or macrovascular (96%) invasion. Most tumors were well-differentiated (64%) and not infiltrative (92%).

**TABLE 5:** Explant pathologic characteristics.

	*n*	LT
Largest diameter, mean (mm) ± SD	25	24.9 ± 11
Sum of diameter, mean (mm) ± SD	23	61.3 ± 32.5
Tumor number, n (%)	25	
1 nodule		3 (12%)
2 or 3 nodules		3 (12%)
≥4 nodules		19 (76%)
Extent of tumor necrosis, n (%)	16	
Complete (no viable tumor) (100%)		1 (6.3%)
Subtotal necrosis (≥90%)		1 (6.3%)
Partial necrosis (≥50% and <90%)		3 (18.8%)
Minimal necrosis (<50%)		9 (56.3%)
No necrosis (0%)		2 (12.5%)
Differentiation grade, n (%)	25	
Well differentiated		16 (64%)
Moderately and poorly differentiated		8 (32%)
Not applicable (complete necrosis)		1 (4%)
Infiltrative HCC, n (%)	25	2 (8%)
Satellite nodules, n (%)	16	4 (25%)
Microvascular invasion, n (%)	25	5 (20%)
Macrovascular invasion, n (%)	25	1 (4%)

### Post-Liver Transplantation Morbidity

Post-transplant complications are presented in Table 6. Median length of hospital stay was 19.5 days (IQR: 15.75–29.5). Eight patients underwent revision surgery (30%), of which four were related to bleeding episodes, two to bowel dehiscence, one to bile leakage and one to wall abscess. Seven bleeding episodes occurred (27%), of which four were graft hematomas, one wall hematoma, one digestive ulcer and one hemoperitoneum. Bile duct stenosis concerned three patients (11%), of which two were treated endoscopically and one required no specific management because of the absence of biological repercussions. Two patients presented with bile leakage. Vascular thrombosis occurred in seven patients (27%) and are detailed in Table 6. One patient underwent re-transplantation for severe ischemic cholangitis related to hepatic artery thrombosis. Acute rejection occurred in four patients. Rejection episodes were moderate for three patients and severe for one patient.

**TABLE 6:** Post-transplant complications (no missing data).

	LT
Length of hospital stay (days)	
Mean ± SD	26.5 ± 17.6
Median (IQR range)	19.5 (15.75–29.5)
Revision surgery, n (%)	8 (30.8%)
Bleeding	4
Bowel dehiscence	2
Bile leakage	1
Wall abscess	1
Bleeding, n (%)	7 (26.9%)
Graft hematoma (SCH/subhepatic)	4 (1/3)
Wall hematoma	1
Digestive ulcer	1
Hemoperitoneum	1
Number of peri-operative packed red blood cells	
Mean ± SD	4.8 ± 6.5
Median (IQR range)	3 (1–5.5)
Bile duct stenosis, n (%)	3 (11.5%)
Thrombosis, n (%)	7 (26.9%)
Hepatic artery thrombosis	3 (11.5%)
Pulmonary embolism	1 (3.8%)
Portal/SMV thrombosis	2 (7.7%)
Renal vein thrombosis	1 (3.8%)
Asymptomatic CMV infection, n (%)	10 (38.5%)
Re-transplantation, n (%)	1 (3.9%)
Acute rejection, n (%)	4 (15.4%)
Sepsis, n (%)	9 (34.6%)

One patient had a severe complication. After declamping, the patient presented hemodynamic instability requiring the introduction of noradrenaline. At wound closure, the patient presented a hypertensive peak with tachycardia, followed by severe hypotension and cardiac arrest. Post-arrest (no flow 0, low flow 3 min), cardiac echocardiography showed biventricular failure. Thoracic CT scan showed a sub-segmental pulmonary embolism which did not explain the severity of the clinical condition. Brain scan and coronary angiography did not show any lesion. Due to the persistence of the cardiac failure, ECMO was implemented. The episode was resolutive and no other cardiovascular complications were noted.

### HCC Recurrence and Survival

**FIGURE 4:**
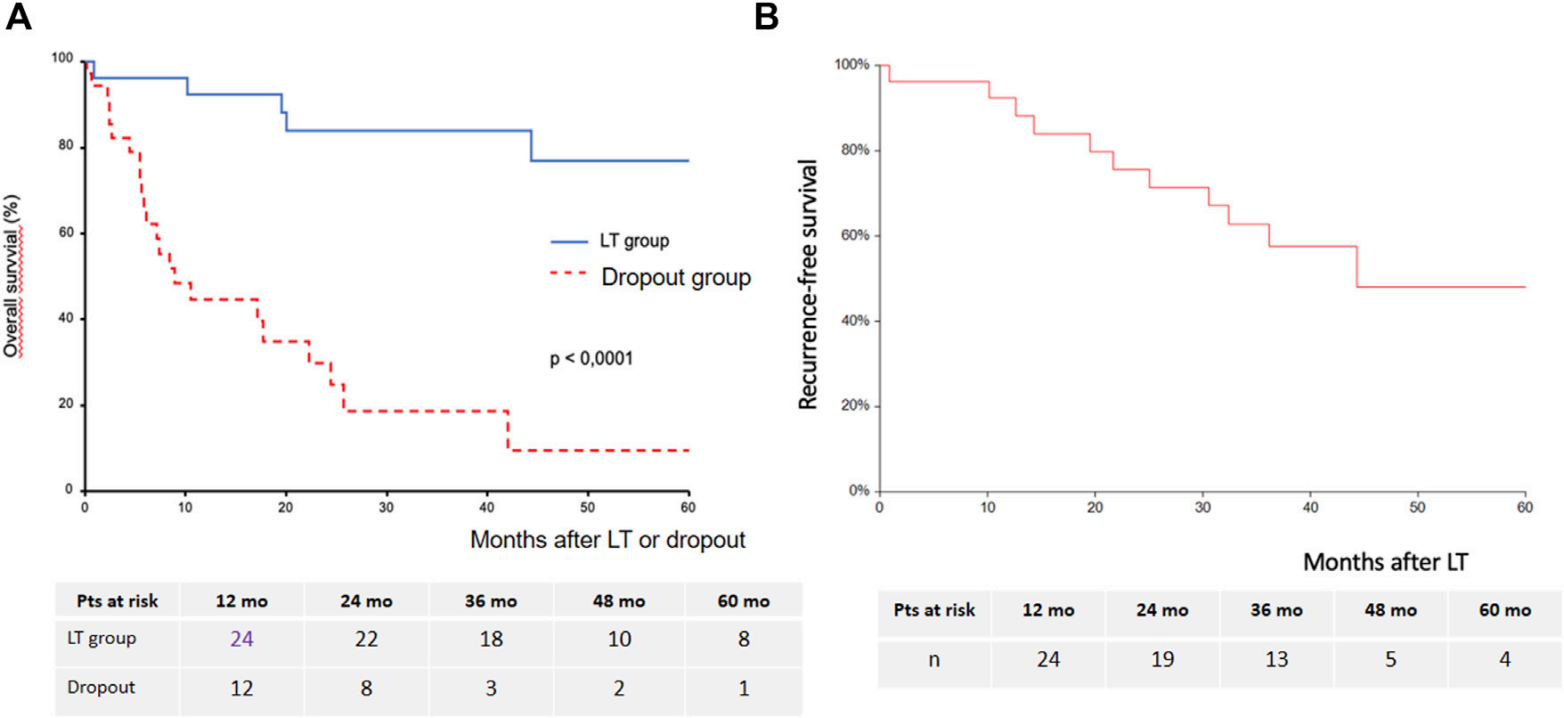
Estimated overall survival after LT or after dropout **(A)** and recurrence-free survival after LT **(B)**.

Mean and median follow-up time were 44.3 ± 24 and 43 months (IQR 28.3–64.9). In the LT group, OS at years 1, 3 and 5 was 96.2%, 83.9% and 76.9%, respectively. In the dropout group, OS at years 1, 3 and 5 was 48.4%, 18.6% and 9%, respectively (Figure 4A). There was a significant difference in OS between the LT group and the dropout group (*p* < 0.0001). The 5-year recurrence-free survival among the transplanted patients was 48% (95% CI: 24%–72%) (Figure 4B). Sorafenib’s indication did not significantly impact OS after LT (data not shown).

**FIGURE 5:**
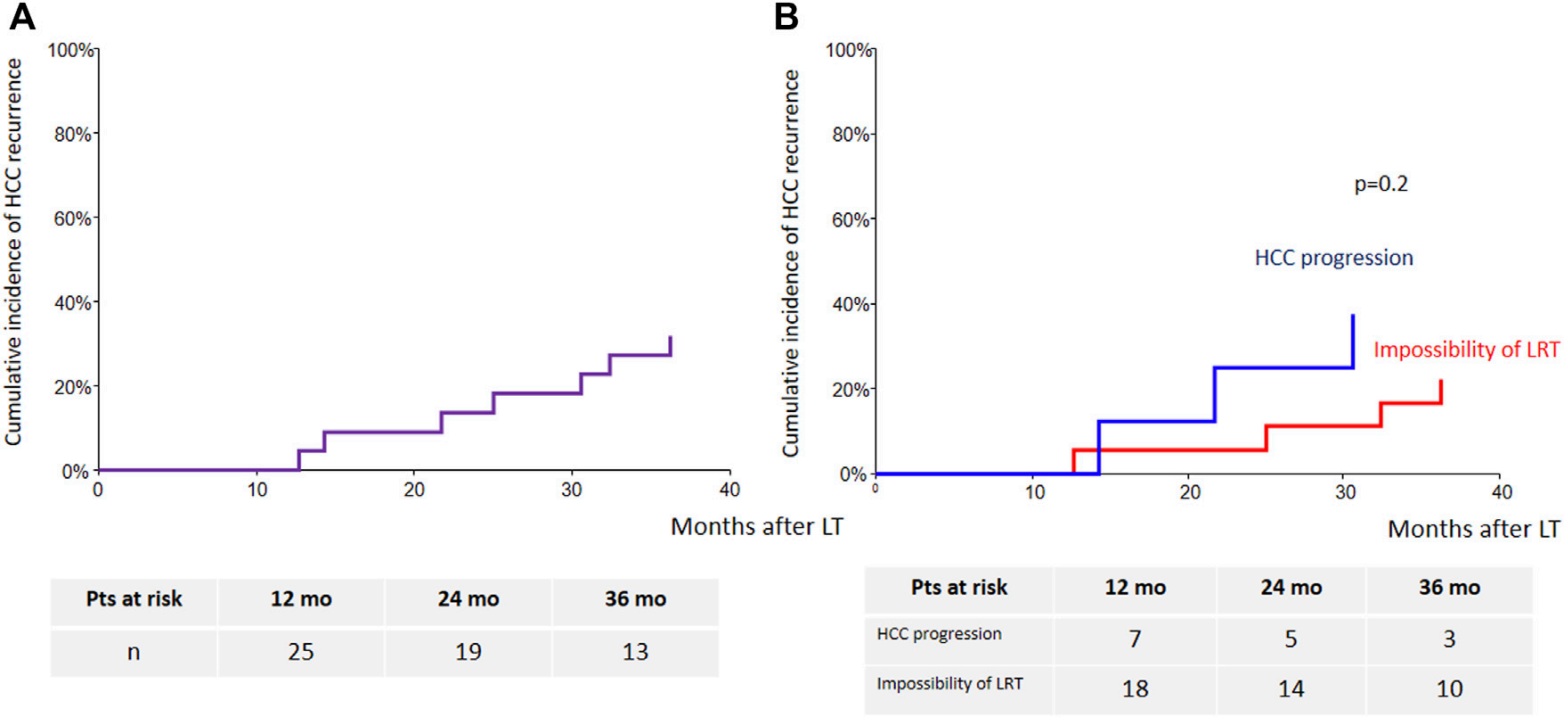
Cumulative incidence of HCC recurrence [**(A)**: whole cohort; **(B)**: according to sorafenib’s indication].

Seven transplanted patients (27% of the LT group) experienced HCC recurrence, which was intrahepatic only for one patient, intrahepatic and extrahepatic for one patient, and extrahepatic for five patients. Extrahepatic tumor recurrence occurred as lung metastases in four patients and lymph nodes metastases in two patients. The mean time to recurrence was 24.7 ± 9 (13–36) months. The 3-year cumulative incidence of HCC recurrence was 32% (95% CI: 17%–59%) (Figure 5A). Sorafenib’s indication was not a predictor of HCC recurrence (Figure 5B).

Demographic, clinical, radiological and explant features were analyzed using linear regression model to identify factors predicting HCC recurrence after LT and are summarized in Table 7. The solely identified factor was the number of HCC within the native liver (HR 1.15, *p* = 0.03). A linear regression multivariable was not performed because of the low number of transplanted patients (*n* = 26) and low number of recurrence (*n* = 7).

**TABLE 7:** Risk factors for HCC recurrence after LT.

	N	HR	95% CI	*p*
Age (years)	26	1	0.95–1.05	0.8
Gender male	26	0.83	0.32–2.1	0.7
Etiology of cirrhosis (alcohol vs. others)	26	1	0.36–2.7	0.9
Indication of sorafenib	26	1.5	0.6–3.5	0.3
MELD	26	1	0.9–1.2	0.4
HCC number at LT	26	1.07	0.8–1.3	0.5
Total HCC diameter at LT	26	1	0.98–1.01	0.7
Unique HCC,				
≤30 mm	26	2.3	0.8–6.3	0.1
AFP-level (UI/L) at listing	26	1	0.99–1	0.2
AFP score at listing	26	1.26	0.8–1.9	0.3
Milan criteria fulfilled at listing	26	0.96	0.4–2.2	0.9
Mean sorafenib start dose	25	1	0.9–1	0.3
AFP score prior to LT	26	2.5	0.5–12	0.2
Last mRECIST radiological response prior to LT	26	0.9	0.55–1.6	0.8
Waiting time from listing to LT	26	1.11	0.9–1.3	0.09
Tumor number on explant	25	1.15	1–1.3	**0.003**
Differentiation grade	25	0.6	0.25–1.5	0.2
Satellite nodules	17	0.9	0.28–2.9	0.8
Microvascular invasion	26	1.4	0.5–3.8	0.5
Re-LT	26	4.47	0.52–38.6	0.25

## Discussion

In the present study, we aimed to analyze natural history and trajectories of patients awaiting LT treated with sorafenib as neoadjuvant therapy, peri-operative morbidity and overall and recurrence-free survival after LT. Twenty-six patients treated with sorafenib (42% of the cohort) underwent LT. Thus, dropout from the waiting list remains a major issue as 58% of our cohort experienced it for tumor progression. Among these patients, half dropped-out after around 8 months (monthly rate of dropout at 3.25% the first year), exceeding the expected average dropout rate of 20% at 12 months according to the Agence de Biomédecine data. In the literature dropout depends on multiple factors, including wait list time, HCC characteristics (solitary tumor greater than 3 cm, two or three tumor nodules), elevated baseline AFP level (≥100 ng/ml), increased AFP concentration, Child-Pugh status, MELD score at listing, use of bridge therapy and response to bridge therapy (20–24). Median waiting time of 12.5 months before LT in our study was consistent with the 12 months median waiting time according to the Agence de Biomédecine data. In our study, there was no significant difference in tumor burden, AFP level or MELD score at listing between the LT and the dropout group which could explain an increase in the dropout rate. Other factors such as tumor biology, genetic signature and escape mechanisms may explain differences in terms of progression on the waiting list. Investigations of the mechanisms underlying the acquired resistance to sorafenib have been led in many studies. One of these mechanisms implicates overexpression of hepatocyte growth factor receptor (HGFR) product of the MET gene which leads to the activation of the Akt and ERK (extracellular signaling-regulated kinase) pathway (25).

Sorafenib failed more frequently to prevent dropout as compared with other studies in a neoadjuvant setting. Truesdale et al. reported that there were no dropout for HCC progression among 10 patients in the sorafenib group of their study (11). Kulik et al. reported the occurrence of disease progression during the trial in only one patient under sorafenib and radioembolization and one patient of the control group (15). Frenette et al. recorded a 20% rate of dropout for tumor progression in their study (12). One explanation for our higher dropout rate may lie in sorafenib treatment indication, which influenced significantly dropout rate. Indeed, patients treated with sorafenib after tumor progression (50% of our cohort) had a significantly higher dropout rate than patients treated with sorafenib because of impossibility of another LRT (multifocal tumor or technical impossibility) (*p* = 0.01). These findings corroborate those of Cuchetti et al. who showed that patients with no response to bridge therapy had the highest dropout rates (23). Our results suggested two different trajectories of natural history which was confirmed by the intention-to-treat survival analysis showing a better survival in patients treated for impossibility of LRT compared to those treated for HCC progression.

The most frequent treatment-related AEs related to sorafenib were dermatological disorders (41.9%), gastrointestinal disorders (37.1%) and fatigue (21%). These results are consistent with the most common events reported in major clinical trials (9, 26). However, these events occurred less frequently in comparison to the safety reports from previous sorafenib monotherapy trials (12, 13). Approximatively half of our cohort started sorafenib at full dose (400 mg twice daily) whereas in other neoadjuvant sorafenib studies, it was initiated at full dose in almost all patients. As a result, we reported fewer dose reductions in our study (41%) than in the other studies. In addition, mean sorafenib treatment time was 6 months, which is higher than findings in other neoadjuvant sorafenib studies where treatment duration ranged from 2.9 to 5.2 months (11–16).

In our cohort, the disease control rate (CR, PR and SD) was 73.2% in transplanted patients. Published series on mRECIST tumor response to TACE prior to LT showed similar rates ranging from 75% to 88% (27–29). Only one study assessed mRECIST tumor response to sorafenib, in combination with TACE (13). This study recorded a disease control rate of 69.5% prior to LT or dropout. One additional point of interest of our study is the well-known underestimation of tumor burden by radiological assessment, compared to histological findings, which is illustrated by the difference in sum of diameter between both evaluations. This notion has been well described in the literature, with rates of tumor under-staging by preoperative imaging ranging between 20% and 40% in most centers (28–31).

Interaction of sorafenib with the transplantation setting is of particular interest for transplant surgeons. High post-LT complication rates have been reported in patients receiving sorafenib before LT (11, 15), but no firm conclusions can be drawn due to the small sample sizes, and other reports showed no increased complication rate (12–14). In our study, the incidence of bile duct stenosis was 11.5% and that of bile leakage was 3.8%. Kulik et al. and Truesdale et al. described both a potentially increased risk for biliary complications of respectively 62.5% and 67% in a sorafenib neoadjuvant setting (11, 15). Our results were in parity with the estimated average rates of the systematic review conducted by Akamatsu in a total of 14,359 liver transplantations, which were of 12% for biliary stricture and 7.8% for biliary leakage (32). Concerning thrombosis, incidence of hepatic artery thrombosis (HAT) was of 3.9% and of 1% for portal vein thrombosis in Duffy et al.’s cohort of 4234 LT recipients (33). In our study, we reported an unexpected higher rate of HAT of 11.5% and of portal vein thrombosis of 7.7%. Among all five (19%) patients who experienced HAT or portal vein thrombosis in our study, three (12%) patients had stopped sorafenib at least 6 months before LT, which makes the impact of sorafenib in the occurrence of thrombosis questionable. Finally, post-operative bleeding was observed in seven (27%) patients, of which four (15%) had continued sorafenib until LT and three (12%) had stopped treatment at least 2 months before LT. When considering only patients having continued sorafenib until LT, these results are below the 20% rate of bleeding leading to revision surgery reported by Schrem and al (34). No pseudo-aneurysm of the hepatic artery were noted in our study, whereas Eilard et al. and Truesdale et al. both recorded respectively a 16.7% and 11.1% rate of pseudo-aneurysm of the hepatic artery. Thus, our study suggests that sorafenib use prior to LT with discontinuation only on the day of transplantation appeared to be safe without increased risk of surgical or transplant-related complications. A case control study could be useful to accurately respond to the question of higher post-LT morbidity in transplanted patients treated with sorafenib.

The rationale for using sorafenib during waiting-list time relies also in its potential to prevent recurrence. In our country, use of AFP score allow to select candidates with a 70% probability of overall survival at 5 years and allows to transplant patients with at low risk of recurrence beyond Milan criteria. Currently, we observe and consider as acceptable a recurrence rate around 15% 5 years following LT. Results of recurrence rates in previous neoadjuvant sorafenib studies were heterogeneous, ranging from 0 to 42%, and impacted by limited sample size (11–15). In our cohort of 26 transplanted patients, seven patients (27%) experienced HCC recurrence, and 15 patients (58%) were alive and free of recurrence at the end of follow-up. However, recurrence free survival close to 50% is questionable in terms of “utility” to transplant such patients, even if new treatments have emerged and give huge benefit in terms of post-recurrence survival. It is important to notice that four patients who had presented HCC recurrence at month 21, 25, 30 and 36 have died more than 5 years after LT (month 64, 66, 83 and 97) which suggest an improvement in the management of HCC recurrence.

This study weakness is the non-randomized design of the study and it is difficult to perceive what would have been the access to LT of patients without sorafenib in the absence of control group. To our knowledge, this is the largest cohort reported to date of use of sorafenib in a neoadjuvant setting. We also recognize that our strategy may appear conflicting with recent guidelines of HCC treatment but neoadjuvant immunotherapy approaches could be associated with significant risks of allograft rejection and such strategy need to be very cautiously explored in dedicated studies.

In conclusion, sorafenib as neoadjuvant treatment provided access to LT for 42% of patients while one- and two-years dropout rates were 39% and 56% (monthly rate of dropout at 3.25% the first year). However, we probably have to separate two different situations of use. Indeed, sorafenib as neoadjuvant treatment can certainly play an important role for patients with impossibility of LRT, as it provides acceptable 5-years intention-to-treat overall and post-LT survivals. Conversely, patients treated for HCC progression presented high dropout rate and low intention-to-treat survival. Thus, it is very questionable in terms of utility with a scarce donor pool, if they should even be considered for still kept listed once the tumor progression has been observed.

## Data Availability

The raw data supporting the conclusion of this article will be made available by the authors, without undue reservation.

## Abbreviations

AE, Adverse event; AFP, Alpha-fetoprotein; CMV, Cytomegalovirus; CI, Confidence interval; CR, Complete response; CT, Computerized tomography; HAT, Hepatic artery thrombosis; HCC, Hepatocellular carcinoma; HFS, Hand-foot syndrome; LRT, Loco-regional therapy; LT, Liver transplantation; MELD, Model for End-stage Liver Disease; MRI, Magnetic Imaging Resonance; OS, Overall survival; PBC, Primary biliary cholangitis, PD, Progressive disease; PR, Partial response; RFA, Radiofrequency ablation; SCH, Subcapsular hematoma; SD, Stable disease/Standard deviation; SMV, Superior mesenteric vein; TACE, Transarterial chemoembolization; TARE, Transarterial radioembolization.
